# The Vacuolar ATPase a2-subunit regulates Notch signaling in triple-negative breast cancer cells

**DOI:** 10.18632/oncotarget.5275

**Published:** 2015-09-25

**Authors:** Sahithi Pamarthy, Mukesh K. Jaiswal, Arpita Kulshreshtha, Gajendra K. Katara, Alice Gilman-Sachs, Kenneth D. Beaman

**Affiliations:** ^1^ Department of Microbiology and Immunology, Rosalind Franklin University of Medicine and Science, North Chicago, IL 60064, USA

**Keywords:** a2V-ATPase, notch signaling, triple negative breast cancer, autophagy, bafilomycin

## Abstract

Triple Negative Breast Cancer (TNBC) is a subtype of breast cancer with poor prognosis for which no targeted therapies are currently available. Notch signaling has been implicated in breast cancer but the factors that control Notch in TNBC are unknown. Because the Vacuolar ATPase has been shown to be important in breast cancer invasiveness, we investigated the role of a2-subunit isoform of Vacuolar ATPase (a2V) in regulating Notch signaling in TNBC. Confocal microscopy revealed that among all the ‘a’ subunit isoforms, a2V was uniquely expressed on the plasma membrane of breast cancer cells. Both a2V and NOTCH1 were elevated in TNBC tumors tissues and cell lines. a2V knockdown by siRNA as well as V-ATPase inhibition by Bafilomycin A1 (Baf A1) in TNBC cell lines enhanced Notch signaling by increasing the expression of Notch1 intracellular Domain (N1ICD). V-ATPase inhibition blocked NICD degradation by disrupting autophagy and lysosomal acidification as demonstrated by accumulation of LC3B and diminished expression of LAMP1 respectively. Importantly, treatment with Baf A1 or anti-a2V, a novel-neutralizing antibody against a2V hindered cell migration of TNBC cells. Our findings indicate that a2V regulates Notch signaling through its role in endolysosomal acidification and emerges as a potential target for TNBC.

## INTRODUCTION

Breast cancer is the most common cancer in women worldwide. The major subtypes of breast cancer are classified based on the presence or absence of hormonal receptors Estrogen (ER), Progesterone (PR) and the Human Epidermal growth factor Receptor (Her2). 15 to 20% of all breast cancers diagnosed are of the Triple-Negative subtype [1]. Triple-Negative Breast Cancers (TNBC) are defined by the lack of expression of ER/PR and Her2 receptors. They are highly aggressive and are more common in younger women and in women of African heritage [2, 3]. Due to the lack of targeted therapies, TNBC has a poor prognosis. Consequently, there is an urgent need to identify new targets to treat patients with TNBC [4].

Notch signaling is an evolutionarily conserved juxtacrine-signaling pathway that regulates cell fate decisions and proliferation. The Notch pathway consists of five ligands of the Delta/Jag/Serrate Family (Dll1, 3 and 4, Jagged 1 and 2) which interact with either of four Notch receptors (Notch 1, 2, 3, and 4). Ligand binding initiates proteolytic cleavage of the Notch receptor by ADAM-TACE enzyme on the plasma membrane followed by S2 cleavage by gamma secretase resulting in the release of the Notch intracellular domain (NICD). NICD is translocated to the nucleus to activate Notch target genes, which include transcriptional repressors of Hes/Hey family among other genes [5]. Notch is required for normal mammary development [6, 7] tumor survival [8] and is dysregulated in breast cancer [9, 10]. Previous studies have highlighted the role of Notch signaling in TNBC [11, 12]. However, the factors contributing to Notch activation in TNBC are still widely unknown. Better understanding of regulation of Notch signaling and its crosstalk with other oncogenic signals in TNBC is essential to design and develop new therapeutic possibilities.

Recent Studies in Drosophila and mammalian cells have identified the V-ATPase as an important regulator of Notch and Wnt signaling [13–18]. By maintaining pH, V-ATPase regulates cellular processes like endocytosis, vesicular trafficking and protein degradation thereby influencing various signaling pathways. In tumors, V-ATPase helps maintain an alkaline intracellular environment favorable for growth and an acidic extracellular environment favorable for invasion [19]. Structurally, the V-ATPase is a multisubunit enzyme complex consisting of a peripheral V1 domain and a transmembrane V0 domain [20]. The V0 domain consists of ‘a’ subunit among others which is expressed as four isoforms: a1, a2 a3 and a4. Increased expression of ‘a’ subunits has been implicated in breast cancer [21, 22]. However, the specific contribution of each isoform towards tumorigenesis is not known. We have previously identified a novel immunomodulatory role of a2V in pregnancy [23, 24] and cancer [25, 26]. a2V is expressed on immune cells [27], regulates apoptosis and innate immune responses during preterm labor [28] and the N terminal domain of a2V (a2NTD) modulates host immune responses in cancer through its involvement in macrophage polarization [29].

In the present study, we report the role of a2V in regulating Notch signaling. a2V is abundantly expressed on the plasma membrane of tumor cells, making it an attractive target for anti-cancer therapies. siRNA mediated knockdown of a2V led to an increase in Notch signaling. Our results show that a2V inhibition blocks autophagic degradation of Notch receptor and thereby increases Notch signaling.

## RESULTS

### a2V is expressed on the plasma membrane of breast cancer cells

**Figure 1:**
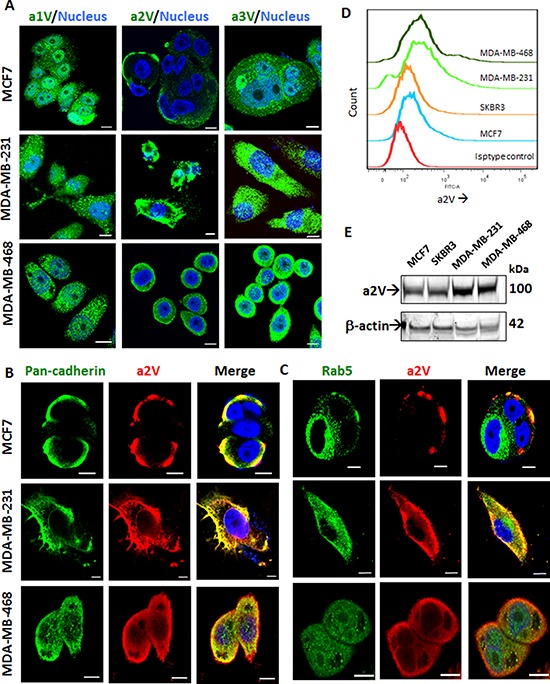
a2V is abundantly expressed on the surface of TNBC cells ER/PR positive MCF7, Triple–negative MDA-MB-231 and MDA-MB-468 cells were grown on chamber slides. Cells were fixed, permeabilized and processed for immunofluorescence microscopy. **A.** Representative images taken with a confocal laser-scanning microscope show expression pattern of V-ATPase ‘a’ subunit isoforms a1, a2, and a3 (green). Cultured cells were stained with antibodies specific for a2V-ATPase (red) along with **B.** plasma membrane marker, pan-cadherin (green) and **C.** early endosome marker, Rab5 (green). Colocalization was examined by confocal microscopy. Representative *z-stack* images are shown. Nucleus was stained with DAPI (blue). Scale bars: 10 μm. **D.** Non-permeabilized Breast cancer cells were cultured, fixed and subjected to surface staining of a2V. Stacked offset histograms are shown. Matched Isotype control from MDA-MB-468 is shown. **E.** Whole cell lysates from MCF7, SkBR3, MDA-MB-231 and MDA-MB-468 were harvested and subjected to Western blot analysis with specific antibody for a2V. β-actin was used as a loading control. DAPI: diamidino-2-phenylindole.

The V-ATPase is a multisubunit enzyme complex that has four defined ‘a’ subunit isoforms of V0 domain - a1, a2, a3 and a4 [20]. In order to determine the role of specific ‘a’ isoforms in TNBC, we studied their expression and cellular localization in ER/PR positive MCF7, HER2-positive SkBr3 and Triple Negative MDA-MB-231 and MDA-MB-468 cell lines. The expression of a1, a2, a3 and a4 isoforms was higher in MDA-MB-231 and MDA-MB-468 as compared to MCF7 reiterating the role of ‘a’ subunit isoforms in cancer invasiveness. The Her2 positive SKBR3 cell line showed low level expression of all four ‘a’ subunits (Supplementary Figure S1A). Confocal microscopy revealed distinct surface expression of a2V. a1 and a3 isoforms showed intracellular punctate staining (Figure 1A). Since a2V showed surface expression in breast cancer cell lines, it was of interest to further identify its specific cellular location. We found distinct colocalization of a2V with plasma membrane marker pan-cadherin (Figure 1B). Recent reports found that mutations within a2V gene caused autosomal recessive cutis laxa type II, highlighting the role of a2V in golgi function [30]. Consistent with the reported localization of ‘a’ subunit isoforms of V-ATPases [20], a2V partially co-localized with early endosome marker Rab5 (Figure 1C) and also with golgi marker Golph4 (Supplementary Figure S2A). We further confirmed the plasma membrane localization of a2V in permeabilized versus non-permeabilized cells by immunofluorescence (Supplementary Figure S2B). Flow cytometric analysis was used for the detection of surface expression of a2V in non-permeabilized cells (Figure 1D). The soluble N-terminus domain of a2V (a2NTD) is cleaved from the a2 subunit of cancer cells and modulates cancer related inflammation [29]. Total a2V was higher in the TNBC cell lines MDA-MB-231 and MDA-MB-468 (Figure 1E) and a2NTD was specifically expressed in these cells (Supplementary Figure S1B). Taken together, our data suggests that a2V is abundantly expressed on the plasma membrane of TNBC cells.

### a2V and Notch1 are upregulated in breast tumors

**Figure 2:**
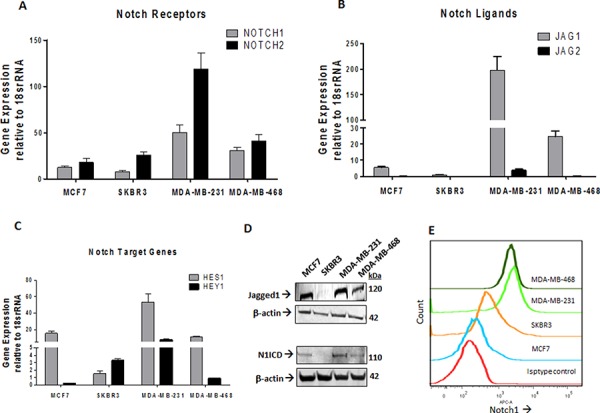
Expressions of Notch1 pathway genes are elevated in TNBC Total RNA extracted from MCF7, SKBR3, MDA-MB-231 and MDA-MD-468 cell lines was subjected to qRT-PCR analysis for mRNA levels of **A.** Notch receptors NOTCH1, NOTCH2 **B.** Notch ligands JAG1, JAG2 and **C.** Notch Target Genes HES1 and HEY1. Data represent mean ± standard error, *n* = 4. **D.** Protein level expression of Notch1 and Jagged 1 in these cell lines was examined by Western blot analysis. β-actin was used as a loading control. **E.** Surface staining of Notch1 in non-permeabilized cells is shown as stacked offset histograms. Matched Isotype control from MDA-MB-468 is shown.

**Figure 3:**
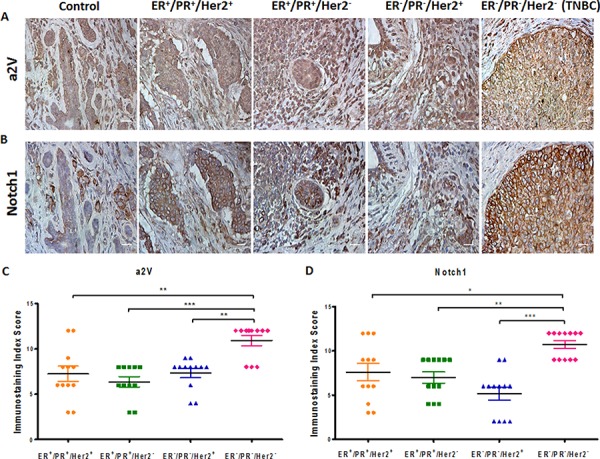
a2V and Notch1 are activated in human breast tumors Tissue microarray containing human breast tumors and normal breast tissues (control) were used to immunolocalize **A.** a2V and **B.** Notch1. Tumors were grouped by receptor-defined subtype. 12 sections per subtype were analyzed. Brown staining - DAB, counterstain - hematoxylin. Original magnification: 400X. Corresponding scatter dot plots show immunostaining index score ((ISIS) = Stained area score (SAS) × Immunostaining intensity score (IIS)) for **C.** a2V and **D.** Notch1. Data represent mean ± standard error, *n* = 12. *P ≤ 0.05, **P ≤ 0.01, ***P ≤ 0.001 between a pair of subtypes. DAB: 3,3′ Diaminobenzidine.

Previous reports have identified that Notch ligand Jagged1 and Notch receptor Notch1 are upregulated in breast cancer [10]. Consistently, we found the gene expressions of Jagged1 (JAG1), NOTCH 1 and Notch target gene HES1 to be upregulated in TNBC cell lines MDA-MB-231 and MDA-MB-468 (Figure 2A–2C). Protein expression of N1ICD and Jagged1 was higher in highly metastatic MDA-MB-231 as compared to low metastatic lines MCF7 and SKBR3 and MDA-MB-231 (Figure 2D). We further confirmed our findings by flow cytometric analysis to detect the surface expression of Notch1 in non-permeabilized cells (Figure 2E). Our results suggest that the Notch1 signaling axis is upregulated in TNBC and correlates with cancer invasiveness. We next investigated the expression of a2V and Notch1 in human breast tumor sections of receptor defined breast cancer subtypes. For this, we examined a tumor tissue array containing 32 human breast tumors representing the four receptor-defined subtypes of breast cancer. Our controls were normal human breast tissues within the same array. Expressions of both a2V and Notch1 appear to be significantly higher in the TNBC intrinsic subtype compared with other subtypes (Figure 3A, 3B). Corresponding immunostaining index scores are shown in Figure 3C, 3D. Together, these results suggest that a2V and Notch1 show higher basal level expression in TNBC.

### a2V inhibition increases Notch signaling in TNBC

**Figure 4:**
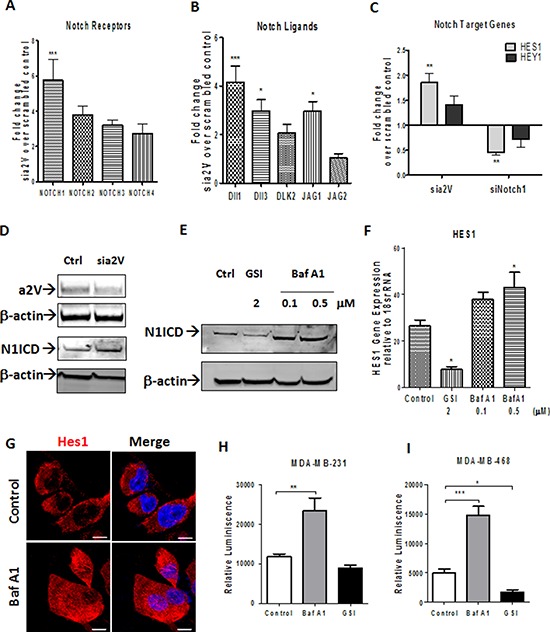
a2V inhibition increases Notch signaling in TNBC **A–D.** TNBC cell line MDA-MB-231 was transfected with siRNA oligonucleotides against a2V or Notch1 along with scrambled control siRNA. Cells were harvested 48 hrs after transfection. Fold change in mRNA expression levels of (A) Notch receptors, (B) Notch Ligands and (C) Notch target genes is shown by qRT-PCR performed on the Notch signaling PCR array. Prior to fold-change calculation, the values were normalized to signal generated from endogenous control 18srRNA. (D) Protein level of Notch1 intracellular domain (N1ICD) following a2V gene silencing is shown by western blot analysis. β actin was used as loading control. **E–I.** MDA-MB-231 cells were treated with Vehicle Control (DMSO), Bafilomycin A1 (Baf A1 – 0.1 or 0.5 μM) or Gamma Secretase Inhibitor (GSI – 2 μM) for 24 hrs. (E) Protein level of Notch1 intracellular domain (N1ICD) following treatment with Baf A1 or GSI is shown by western blot. β actin was used as loading control. (F) Gene expression expression levels of Hes1 relative to endogenous control 18srRNA is shown. (G) Hes1 protein expression is shown by immunofluorescence (H and I). Independently, MDA-MB-231 and MDA-MB-468 were transfected with a RBP-j Notch reporter construct and then treated with Vehicle control, 0.5 μM BafA1 or 2 μM GSI for 24 hrs. Notch reporter levels in (H) MDA-MB-231 and (I) MDA-MB-468 as measured by luciferase assay. Data represent mean ± standard error, *n* = 4. *P ≤ 0.05, **P ≤ 0.01, ***P ≤ 0.001 compared to control. RBPj: Recombinant Binding Protein Suppressor of Hairless.

Endocytosis and endosomal transport of both Notch receptors and its ligands modulates Notch signaling [31]. Because we found colocalization of a2V on plasma membrane and early endosomes, we hypothesized that a2V regulates Notch signaling in TNBC. To test our hypothesis, we performed siRNA-mediated knockdown of a2V in TNBC cell line MDA-MB-231, which demonstrated robust a2V expression and Notch signaling activity (Figures 1 and 2). The efficiency of 3 individual a2V siRNA treatments relative to controls was assessed using RT-PCR and the most efficient siRNA was selected for further experiments (Supplementary Figure S3A). We also confirmed that genetic knockdown of a2V was specific and did not lead to compensatory up-regulation of other ‘a’ subunit isoforms (Supplementary Figure S3D). To investigate if Notch signaling pathway was activated in response to genetic knockdown of a2V, we assessed the mRNA expression of an array of 48 genes involved in Notch signaling. a2V knockdown led to a significant increase in mRNA expression of NOTCH1, Dll1 and JAG1. Other Notch receptors (NOTCH 2–4) and Notch ligands (DLK2, JAG2) also showed an increase (Figure 4A, 4B). Next, using Notch1 siRNA as a reference control, we examined the expression of Notch target genes HES1 and HEY1 of which, we found HES1 to be significantly upregulated (Figure 4C). Efficiency of Notch1 siRNAs is shown in Supplementary Figure S3B, S3C. However, expression of genes involved in Notch receptor processing, transcription factors and cofactors remained unaffected (Supplementary Table S1). We further confirmed our findings by treating TNBC cells with Bafilomycin (Baf A1), a known specific inhibitor of V-ATPase. Treatment with a2V siRNA or Baf A1 led to an increase in N1ICD, the main downstream effector of Notch signaling. Gamma Secretase inhibitor (GSI) a known inhibitor of the Notch pathway served as a reference control (Figure 4D, 4E). Corresponding to increase in NICD, increased mRNA expression and nuclear accumulation of HES1 was observed (Figure 4F, 4G). Further, we employed a Notch RBPj reporter construct to measure Notch pathway activity. Baf A1 treatment showed a significant increase in Notch reporter activity of MDA-MB-231 and MDA-MB-468 cells as measured by luciferase assay (Figure 4H, 4I). Together, these results demonstrate that a2V-ATPase inhibition by siRNA as well as Baf A1 leads to an increase in Notch signaling in TNBC.

### BafA1 increases Notch signaling by altering autophagic flux

**Figure 5:**
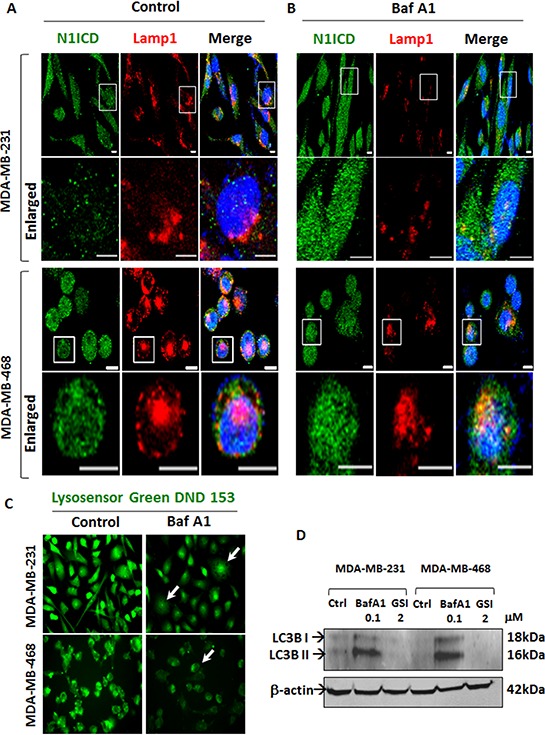
V-ATPase inhibition blocks Notch receptor degradation by inhibiting lysosomal acidification and autophagy TNBC cells were grown on chamber slides and treated with vehicle control or 0.1 μM Baf A1 for 4 hrs. Cells were fixed, permeabilized and processed for immunofluorescence microscopy. Localization of N1ICD (green) and Lamp1 (red) in **A.** control or **B.** Baf A1 treated MDA-MB-231 and MDA-MB-468 cells by immunofluorescence. Nucleus was stained with DAPI (blue). Scale bars: 10 μm. **C.** Lysosensor Green DND 153 staining of acidic intracellular compartments in MDA-MB-231 and MDA-MB-468 cells treated with control or Baf A1. Representative images for each cell line are shown. **D.** MDA-MB-231 and MBA-MB-468 cells were treated with vehicle Control, 2 μM GSI or 0.1 μM Baf A1 for 24 hrs and immunoblotted for LC3B. β actin was used as loading control.

Sorting of Notch receptor to early or late endosomal compartments determines the positive or negative effect of endocytosis on Notch signaling [32]. Based on this understanding and our recent report on the role of a2V in autophagy [33], we reasoned that V-ATPase inhibition in TNBC increases Notch signaling by blocking Notch receptor degradation in lysosomes. Baf A1 is known to hinder autophagosome maturation by inhibiting fusion with lysosomes [34]. To investigate this possibility, we examined the expression of LAMP1 and LC3B, markers of lysosome and autophagosome respectively. In BafA1 treated cells, lysosomes were disintegrated and Lamp1 expression was decreased. Correspondingly, cytoplasmic accumulation of N1ICD was observed (Figure 5A, 5B). BafA1 disrupted endolysosomal acidification and induced autophagic vacuolization, as seen by the significant reduction in Lysosensor, a fluorescent acidophilic dye that incorporates in acidified vacuoles (Figure 5C, white arrows). Further, we found accumulation of both LC3B-I and LC3B-II following treatment with Baf A1, demonstrating inhibition of autophagic flux. Notch inhibitor GSI had no effect on autophagy (Figure 5D). These results suggest that V-ATPase inhibition affects endolysosomal acidification and increases Notch signaling by blocking autophagic Notch receptor degradation.

### V-ATPase inhibition increases β-catenin

**Figure 6:**
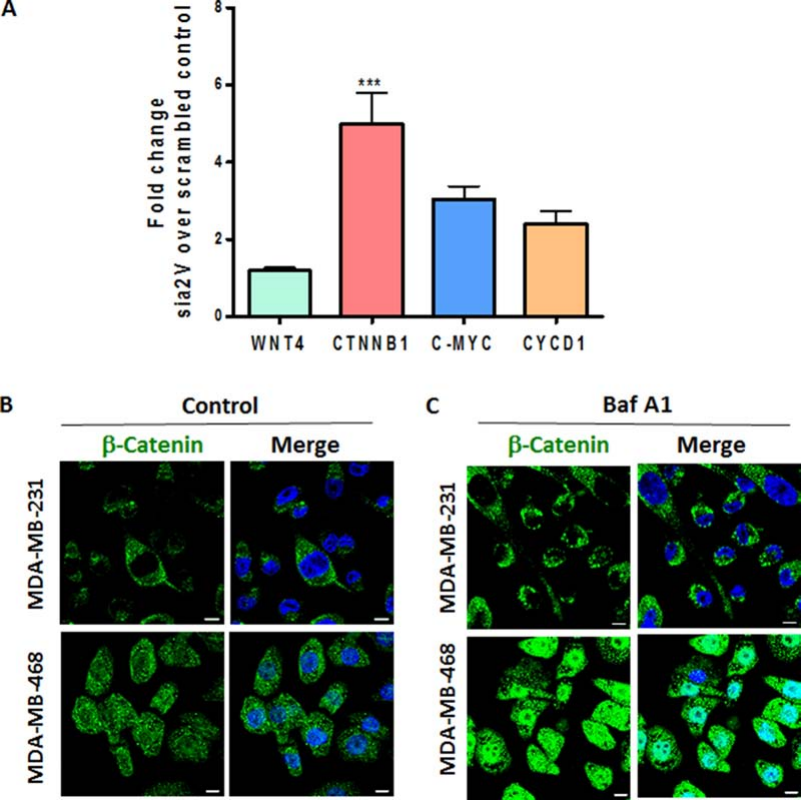
a2V-ATPase inhibition enhances Wnt signaling in TNBC **A.** MDA-MB-231 cells were transfected with scrambled control or a2V siRNA and harvested after 48 hrs of transfection. Fold change in mRNA expression levels of Wnt signaling genes WNT4, β-catenin (CTNNB1), C-MYC and Cyclin D1 (CYCD1) was assessed by qRT PCR. Prior to fold--change calculation, the values were normalized to signal generated from endogenous control 18srRNA. Data represent mean ± standard error, *n* = 4. *P ≤ 0.05, **P ≤ 0.01 compared to control siRNA. (B and C) TNBC cells were grown on chamber slides and treated with **B.** vehicle control or **C.** 0.1 μM Baf A1 for 4 hours. Cells were fixed, permeabilized and processed for immunofluorescence microscopy. Localization of β-catenin (green) is shown. Nucleus was stained with DAPI (blue). Scale bars: 10 μm.

Cytoplasmic localization of β-catenin results in poor prognosis of breast cancer [35, 36]. Our findings on Notch activation prompted us to investigate the role of V-ATPase inhibition on Wnt pathway in TNBC, since both pathways cooperate in mammary tumorigenesis and are affected by autophagy [37, 38]. a2V knockdown led to an increase in mRNA expression levels of β-catenin (CTNNB1), C-MYC and Cyclin D1 (CYCD1) in MDA-MB-231 cells (Figure 6A). Immunofluorescence staining of TNBC cells demonstrated that β-catenin expression is increased following V-ATPase inhibition (Figure 6B, 6C). Therefore, the influence of V-ATPase inhibition on Wnt signaling pathway was consistent with our results obtained on Notch signaling pathway

### a2V inhibition decreases cell viability and migration of TNBC cells

**Figure 7:**
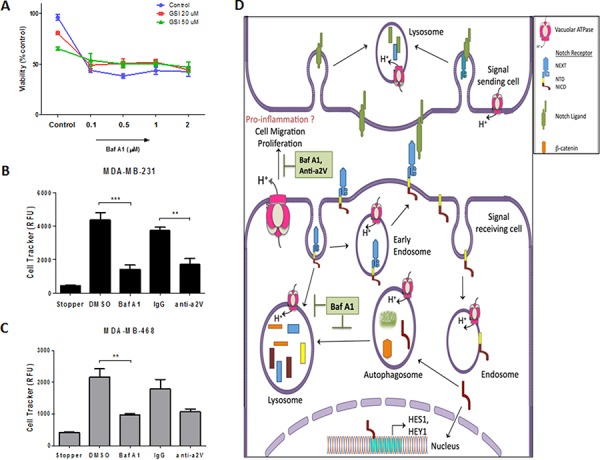
Effect of V-ATPase inhibition on cell viability and migration of TNBC cells MDA-MB-231 and MDA-MB-468 cells were seeded (1 × 10^4^ cells/well) in 96 well plates and treated with vehicle control, Baf A1 or GSI as indicated at various concentrations for 48 hrs. **A.** Cell viability measured by ApoTox-Glo™ triplex assay is shown as percent control. (B, C) Effect of V-ATPase inhibition on cell migration was demonstrated by using Oris™ Cell Migration Assay. 5 × 10^4^ cells were plated in 96 well plates with stoppers. When adherent, stoppers were removed and cells were treated with 1 μM Baf A1, 10 μg/mL anti-a2V or their respective controls DMSO or IgG and incubated for 48 hrs to permit cell migration. Quantified fluorescence of Cell Tracker Green CMFDA in the detection zone of **B.** MDA-MB-231 and **C.** MDA-MB-468 is shown. Data represent mean ± standard error, *n* = 3. *P ≤ 0.05, **P ≤ 0.01, ***P ≤ 0.001 compared to vehicle control. **D.** Summary: V-ATPase regulates Notch signaling in TNBC through its role in endolysosomal acidification and autophagy. Following interaction with ligands on signal sending cell, the Notch receptor on signal receiving cell is endocytosed and cleaved resulting in the release of NICD, which translocates to the nucleus and activates Notch target genes HES1 and HEY1. V-ATPase inhibition by Baf A1 or a2V knockdown halts the recycling (early endosomes) and degradative (autophagosome-lysosomes) routes of Notch receptor, thereby accumulating NICD and resulting in enhanced signaling. Furthermore, V-ATPase inhibition induces apoptosis and hinders cell migration of TNBC cells.

With their effect on autophagy and apoptosis, V-ATPase inhibitors are promising drug targets in cancer [39–41]. Previous studies have reported the cell proliferative role of Notch signaling in cancer [8]. In this view, our finding of Notch activation following Baf A1 treatment prompted us to investigate the effect of combinatorial treatment with Baf A1 and Notch signaling inhibitor GSI in TNBC. Baf A1 treatment resulted in a significant decrease in TNBC cell viability and increase in cytotoxicity through apoptosis as measured by the ApoToxGlo triplex assay. However, GSI by itself or in combination with Baf A1 did not cause significant change in cell viability (Figure 7A) (cytotoxicity and apoptosis data not shown). Treatment with Concanamycin A, another known V-ATPase inhibitor also caused a significant decrease in cell viability (Supplementary Figure S4A). These results suggest that V-ATPase inhibition is cytotoxic to TNBC cells independent and regardless of its effect on Notch pathway. We have recently shown that a2V inhibition delays tumor growth in the 4T1 TNBC mouse xenograft model [42]. To investigate the specific role of a2V in TNBC cell migration we treated MDA-MB-231 and MDA-MB-468 with Baf A1 or anti-a2V, a novel neutralizing antibody against a2V. Since anti-a2V can only inhibit the activity of plasmalemmal a2V, no significant effect on cell viability was observed (Supplementary Figure S4B). However, V-ATPase inhibition in general and a2V inhibition in particular significantly hindered TNBC cell migration (Figure 7B, 7C). Representative images of migration zone are shown in Supplementary Figure S4C. Taken together, our data suggests that a2V inhibition attenuates the migration of invasive cancer cells, thereby making it an attractive therapeutic for TNBC.

## DISCUSSION

In this study we identified novel evidence of a link between Notch and V-ATPase pathways in TNBC. a2V is abundantly expressed on the plasma membrane of breast tumor cells. a2V inhibition enhanced Notch signaling in TNBC by blocking autophagic degradation of NICD. Finally, a2V-ATPase inhibition by Baf A1 or anti-a2V significantly attenuated tumor cell viability, increased apoptosis and attenuated cancer cell migration (Figure 7D).

V-ATPase is a multi subunit proton pump responsible for maintaining intracellular and extracellular pH. Previous results have identified the role of a3 and a4 subunits in breast cancer invasiveness [21, 22]. Here, we find that a2V shows abundant plasma membrane expression in breast tumors and TNBC cell lines and regulates TNBC cell migration. The distinct outcomes from targeting specific ‘a’ subunit isoforms could be attributed to their cellular distribution. Studies suggest that V-ATPase on the plasma membrane is involved in the acquisition of a metastatic phenotype [43]. Our findings lend further support to our recent results demonstrating that a2V is accumulated on the surface of ovarian tumors, associates with invasion assembly related proteins and plays a critical role in tumor invasion by modulating the activity of matrix degrading proteases [44]. a2V is therefore a distinct biomarker and therapeutic target in a large variety of cancers.

Notch signaling plays an important role during development and tumorigenesis. Accumulating evidence supports the association of Notch activation with TNBC tumor development [45, 46]. Consistently, we found increased expression of the receptor Notch1 in human breast tumors as well as cell lines of the TNBC subtype. Canonical Notch signaling occurs as a result of the interaction between Notch receptors and their ligands, both of which require vesicular trafficking and endocytosis for maturation, signaling, recycling, and degradation processes [47]. In TNBC, a2V is distinctly expressed on the plasma membrane and on early endosomes indicating a possible role of a2V in the receptor endocytosis pathway.

The retention of Notch in endosomal vesicles accelerates its cleavage and intensifies Notch signaling, as seen in flies with mutations in the Endosomal Sorting Complexes Required for Transport (ESCRT) machinery [48]. In line with these reports, our results show that a2V inhibition increases Notch signaling. However, previous studies have identified V-ATPase to be required for Notch signaling in drosophila and mammalian cell lines [14–18]. In contrast, our findings suggest a negative regulatory role for a2V on Notch signaling. These distinct outcomes could be attributed to the fact that endocytosis is required not only for the activation of internalized receptors but also for degradation of the receptor in lysosomes [49, 50]. In fact, it has been shown that Bafilomycin does not affect initial ligand internalization or recycling pathway but selectively affects the degradative pathway by preventing cargo delivery from early to late endosomes [51]. We speculate that endosomal sorting of Notch to different vacuolar compartments is important in determining the positive or negative influence of V-ATPase on Notch signaling.

Our results with autophagic Notch receptor degradation are in line with these studies as well as studies showing Notch activation in response to autophagy inhibition [52, 53]. Therefore, by halting the degradative, and recycling routes, V-ATPase inhibition leads to a pause in normal endosomal processing of Notch, which in turn accumulates NICD and enhances Notch signaling. Autophagy has been shown to negatively regulate the Notch and Wnt pathways [38, 54, 55]. Notably, we found that V-ATPase inhibition leads to upregulation of β-catenin, the central downstream effector of the Wnt pathway. Our data shows that a2V could influence both Wnt and Notch signaling pathways by accelerating the degradation of β-catenin and NICD respectively. Previous studies have highlighted the role of Baf A1 in inhibiting autophagy and initiating apoptotic cell death [40]. In line with these reports, we find that V-ATPase inhibition markedly reduces cell viability in TNBC by inducing apoptosis. Owing to the negative regulation of Notch signaling by V-ATPase, we found no synergy between inhibitors for both molecules. Although it is known that V-ATPase regulates the signaling machinery responsible for tumor cell migration [56], for V-ATPase inhibition to be effective in tumors, the particular subunit isoform abundantly expressed in each tumor type should be identified and specifically targeted [57]. Notably, we show that anti-a2V a novel-neutralizing antibody slows cell migration. In this view, a2V emerges as a novel therapeutic that can be used in combination with other existing therapies for TNBC.

Blocking of endolysosomal acidification via V-ATPase silencing or inhibition altered the autophagic flux and led to enhancement of Notch signaling beyond its basal level. Although, the role of Notch signaling in breast cancer is thought to be primarily pro-tumorigenic, we observed that the activation of Notch in response to V-ATPase inhibition in TNBC did not lead to increase in cell survival. We speculate that the increase in Notch signaling is part of an inflammatory response mediated by V-ATPase. Studies have highlighted the role of Notch in upregulating pro-inflammatory factors that influence macrophage polarization and more specifically, its association with activated NF-κ B, the master immune regulator [58, 59]. We have previously reported that during infection induced preterm labor, reduction of a2V simultaneously activated apoptosis and inflammatory responses thereby leading to preterm labor [28]. Based on this understanding, we believe that a2V regulates anti-tumoral responses by activating Notch signaling, thereby stimulating pro-inflammatory factors and finally leading to cell death.

Taken together, the results from this study demonstrate that a2V regulates Notch signaling in breast tumor cells through its role in endolysosomal acidification. An important next step would be to decipher the mechanisms by which a2V mediated Notch upregulation would contribute to cancer related inflammation.

## MATERIALS AND METHODS

### Cell culture and reagents

MCF7, SK-BR3, MDA-MB231 and MDA-MB-468 cell lines were obtained from the American Type Culture Collection (Manassas, VA, USA). MCF7 was cultured in MEM medium (Gibco – Life Technologies, Carlsbad, CA, USA) containing a final concentration of 0.01 mg/ml human recombinant insulin. SK-BR3 was cultured in MCoy 5A medium (Hyclone, UT, USA). MDA-MB-231 and MDA-MB-468 were cultured in MEM medium (Gibco). All media were supplemented with 10% fetal bovine serum, 2 mM L-glutamine, 100 units/mL penicillin G, and 100 μg/mL streptomycin sulfate and grown in a humidified incubator at 37°C and 5% CO_2_ atmosphere. Bafilomycin A1 (Millipore, Darmastabt, Germany), Concanamycin A (Sigma-Aldrich, St. Louis, MO, USA) and Gamma Secretase inhibitor (GSI-IX, Millipore) were dissolved in DMSO and used at the indicated concentrations.

### RNA isolation and reverse transcription-PCR

Total RNA was extracted by Trizol^®^ Reagent (Ambion - Life Technologies) and reverse transcribed into cDNA using first strand cDNA synthesis kit (Roche Diagnostics, Mannheim, Germany). Duplex RT-PCR was performed using StepOnePlus™ Real-time PCR (Applied Biosystems - Life Technologies) with 10μl reaction volume and 18srRNA as the internal reference. Prevalidated commercial primers for ‘a’ subunit isoforms ATP6V0a1, ATP6V0a2, ATP6V0a3, ATP6V0a4; Notch pathway genes NOTCH1, NOTCH2, JAG1, JAG2, HES1, HEY1; Wnt pathway genes CTNNB1, c-MYC, WNT4, CYCD1 and internal control *18srRNA* were purchased from Applied Biosystems. Universal fast PCR Master Mix reagent (Applied Biosystems) was used for qPCR amplification of the cDNA.

### PCR gene array

The mRNA expressions of 44 Notch pathway genes was profiled by Taqman PCR Array for Human Notch signaling pathway (Applied Biosystems - 4414165) according to the manufacturer's instructions. RT-PCR was performed in 96-well plate format using the ABI 7500 Real-Time PCR System. Fold changes relative to control samples were calculated using the ΔΔC_t_ method. ΔΔC_t_ values from each sample were normalized by four housekeeping genes, which did not change across the conditions (18s, GAPDH, HPRT1, ACTB). A threshold of 1.5 was used to identify genes of interest.

### Antibodies

Antibodies raised against the V-ATPase ‘a’ subunit isoforms a1 and a2 were generated in our laboratory. The mouse anti-a2V neutralizing antibody against 488–510 amino acids of trans-membrane region (Antibody clone 2C1) and rabbit anti-a2NTD against N terminal domain (Antibody Clone 470) were used as described previously [25, 44, 60]. Anti a1 antibody was raised in rabbit against the synthetic peptides from unique regions of a1 (amino acids 73–95; RKANIPIMDTGENPEVPFPRD) by Covance (USA) and anti a3 antibody was purchased from Abnova, USA. Notch1 (antibody clone EP1238Y) and organellar markers Rab5, Pan-Cadherin and Golph4 were from Abcam. For Western Blot we used cleaved Notch1 antibody Val1744 (Cell signaling, Danvers, MA), Jagged1 (Antibody clone H114, Santa-Cruz, CA), LC3B (Abcam). β-actin (antibody clone AC-74) was purchased from Sigma Aldrich and used as the loading control. For immunohistochemistry we used Notch-1(Santa-Cruz, antibody clone C-20), and anti-a2V. For flow cytometry we used Notch1-APC (Biolegend, San Diego, CA) and FITC conjugated anti-a2V (Covance, Princeton, NJ).

### Immunofluorescence and lysosensor assay

Breast cancer cell lines were plated in 8-well chamber slides (Nunc, USA) at 1 × 10^4^ cells/well and were allowed to adhere overnight. Cells were washed with PBS, fixed for 15 min with 4% paraformaldehyde, and permeabilized with 0.1% Triton X-100 for 10 min. Nonspecific sites were blocked for 1 hr at room with 3% BSA and incubated with primary antibodies, washed 3 times in PBS and visualized with Alexa Fluor^®^ 488 or Alexa Fluor^®^ 594 (Invitrogen) labeled antibodies. For confocal microscopy, the stained cells were imaged on an Olympus Fluoview Fv10i confocal microscope. Analysis was performed using Fv10i Flouview Ver.3.0 software. For Lysosensor assay, cultured cells were incubated with or without Bafilomycin A1 (Millipore) for 30 min in HBSS containing 10 mM HEPES. The cells were then loaded with LysoSensor Green DND-153 (1 μM; Molecular Probes, Life Technologies, Carlsbad, CA) for 15 min at 37°C, washed twice with PBS and immediately visualized with a Nikon eclipse TE2000-S florescence microscope (Nikon Instrument INC).

### Immunohistochemistry

Paraffin embedded human breast cancer and corresponding normal breast tissue sections were obtained from Biochain Institute, Inc (Newark, CA). For antigen retrieval, sections were boiled in sodium citrate buffer (pH = 6). Immunohistochemical staining of Notch1 and a2V was carried out using a method based on horseradish peroxidase-labeled polymer (EnVis ion+ Dual Link System-HRP; DAKO) according to manufacturer's protocol. The sections were counterstained with Mayer's hematoxylin and mounted in Faramount aqueous mounting medium (Dako) and evaluated by light photomicroscopy (Carl Zeiss, Weesp, The Netherlands). Tissue immunostaining was quantified according to the method described in [61]. Briefly, scores were assigned based on 2 parameters; percent of stained area (<25% = 1, 25–50% = 2, 50–75% = 3, >75% = 4) and staining intensity (0 = No staining, 1 = Weak, 2 = Moderate, 3 = Strong). The total Immunostaining Index Score (ISIS) was generated by using the following equation: stained area score (SAS) multiplied by the immunostaining intensity score (IIS): (ISIS = SAS × IIS).

### Protein extraction and immunoblotting

Cells were harvested, washed with ice-cold PBS and lysed in Nonident P-40 (NP-40) lysis buffer containing protease and phosphatase inhibitors (Pierce Protein Biology, USA). Protein concentration in the lysate was determined by BCA protein assay (Thermo Scientific, Rockford, IL, USA). The proteins were separated by 4–12% SDS-PAGE and blotted onto PVDF transfer membranes. The membranes were blocked at room temperature for 1 hr in 5% nonfat dry milk in TBS-T followed by incubation with the indicated primary antibody in TBST containing 5% nonfat milk overnight at 4°C. The membranes were washed three times for 10 min with TBST, followed by incubation with Donkey anti-rabbit IRDye-800CW, or Donkey anti-mouse IRDye-680CW secondary antibodies (LI-COR Bioscience, Lincoln, NE) in TBST containing 3% nonfat milk for 1 hr at RT. Fluorescent blots were imaged by Odyssey Infrared Imaging System (LI-COR Biosciences).

### Flow cytometry

To evaluate the surface expression of a2V and Notch1, breast cancer cells were stained similar to immunofluroescence protocol with a2V and Notch1 antibodies and matched isotype-control (Biolegend). Stained cells were counted on a BD LSR II flow cytometer (BD Biosciences). Data was analyzed with FlowJo software (Tree star, Ashland, OR).

### RNA interference

siRNA pools specific for a2V, Notch1 and scrambled control were purchased from Origene (Rockville, MD, USA). Briefly, 3 × 10^5^ cells were plated in six well plates. Cells were transfected with siRNA (final concentration, 10 nM) using lipid-mediated transfection with Lipofectamine RNAiMAX (Invitrogen) according to the manufacturer's instructions. For all experiments, data were collected 48 hrs post-transfection.

### Luciferase reporter assay

Cignal Lenti RBP-Jk reporter was obtained from SABiosciences (Frederick, MD, USA), which was a ready-to-transduce RBP-Jk-responsive firefly luciferase reporter for monitoring the Notch signaling pathway activity. Cells were seeded at 1 × 10^4^ cells/well in 96-well plates. The following day cells were transduced with 5 × 10^5^ TU of RBP-Jk Reporter Assay (luc) or Cignal Lenti Negative Control in medium without antibiotics and serum. After 48 hrs, transduced cells were exposed to required treatments and luciferase activity was measured with the Dual-Luciferase Reporter Assay System (Promega, Madison, WI, USA), according to the manufacture's instructions.

### Cell viability, apoptosis and migration assays

Briefly, cells were trypsinized to seed at a density of 5 × 10^3^ to 1 × 10^4^ cells/well in 96-well plates. After 48 hours, cells were subjected to the indicated treatments and culturing periods. The following assays were performed according to the manufacturer's instructions. ApoTox-Glo™ Triplex Assay (Promega) was performed to assess viability, cytotoxicity and caspase-3/7 activation within a single assay well. The migration of cells was examined using the Oris™ Cell Migration Assay kit (Platypus Tech, Fitchburg, WI, USA). Relative absorbance, fluorescence or luminescence from the assays was detected using a Multi-Detection Microplate Reader (Synergy HT, Biotek Instruments, USA).

### Statistical analysis

All experiments were conducted at least in triplicates. Results are expressed as mean value of SEM. Treatments were compared to controls by Student's *t*-test or one-way ANOVA followed by Dunnets post hoc test using GraphPad Prism 5.0 software. Differences between groups were considered statistically significant at *P* < 0.05.

## SUPPLEMENTARY FIGURES AND TABLE


