# Supplementary material for: The Vacuolar ATPase a2-subunit regulates Notch signaling in triple-negative breast cancer cells
Source: Oncotarget. 2015 Sep 25;6(33):34206–20. doi: 10.18632/oncotarget.5275 (PMC4741446; doi:10.18632/oncotarget.5275)
Supplement: Supplementary file 1 [file oncotarget-06-34206-s001.pdf]

SUPPLEMENTARY FIGURES AND TABLE

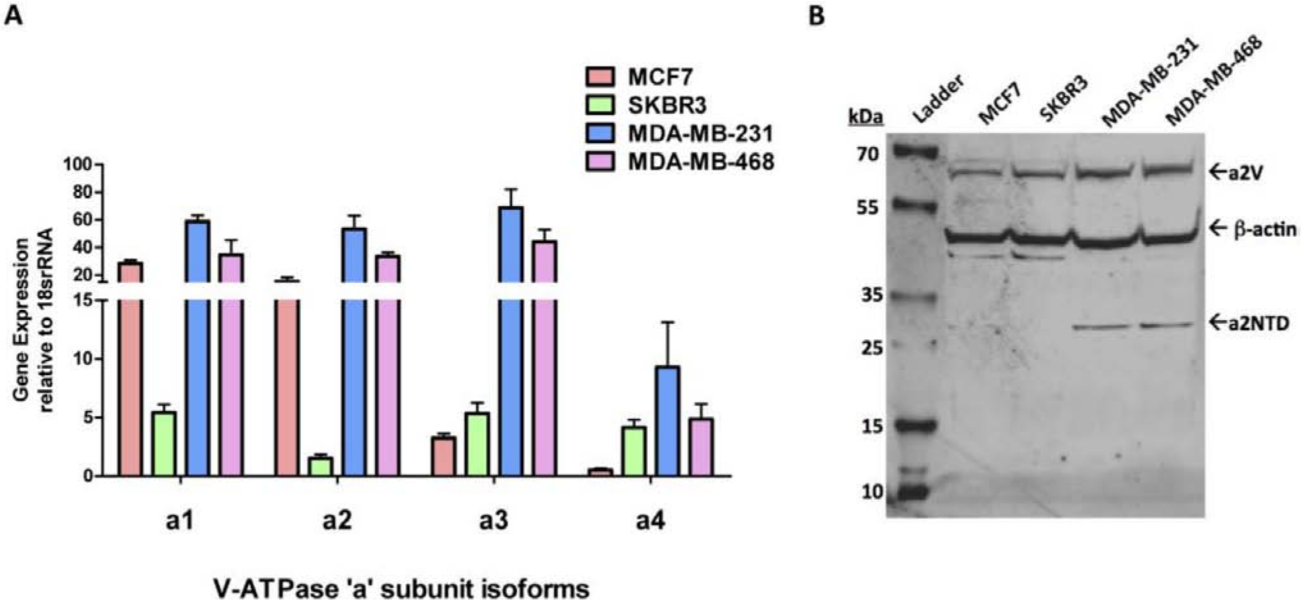

Supplementary Figure S1: Expression of 'a' subunit isoforms in breast cancer cells. A. Total RNA was extracted from ER/PR positive MCF7, HER2-positive SkBr3 and Triple–negative MDA-MB-231 and MDA-MB-468. The expression of a1, a2, a3 and a4 subunits of V-ATPase was analyzed by qRT PCR. mRNA expression relative to endogenous control 18srRNA is shown. Data represent mean ± standard error. **B.** Whole cell lysates from MCF7, SkBR3, MDA-MB-231 and MDA-MB-468 were harvested and subjected to Western blot analysis with an antibody for a2NTD. β-actin was used as a loading control.

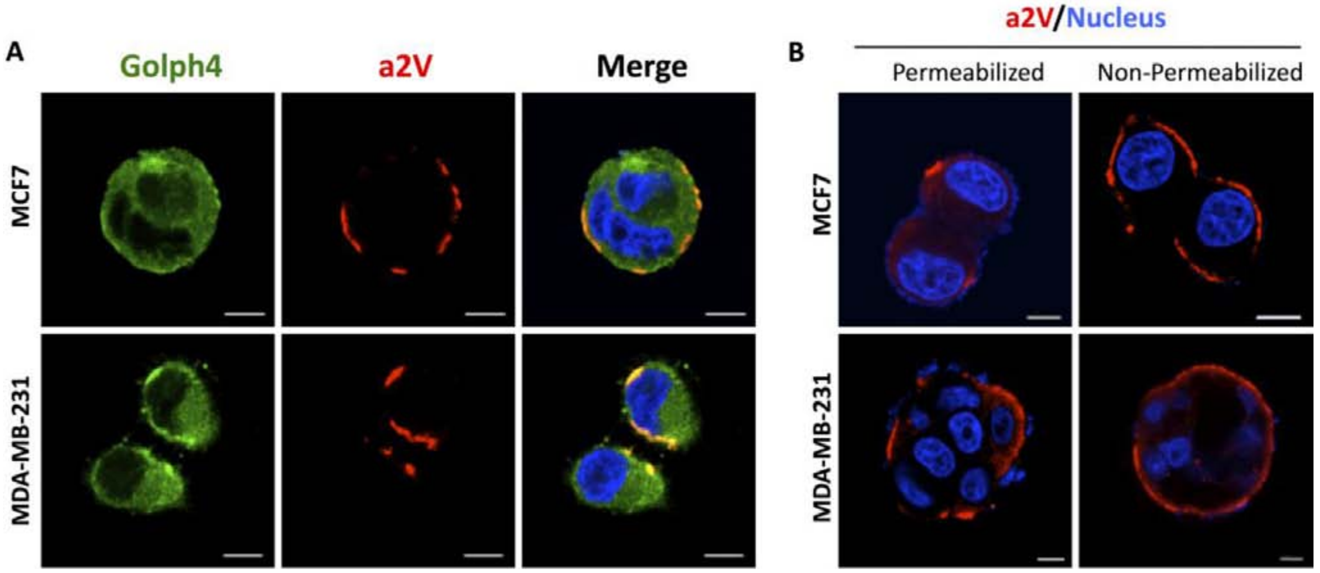

Supplementary Figure S2: Colocalization of a2V with Golgi. MCF7 and MDA-MB-231 cells were grown on chamber slides. Cells were fixed, permeabilized and processed for immunofluorescence microscopy. **A.** Representative images show cells were stained with antibodies specific for a2V-ATPase (red) along with golgi marker Golph4 (green). Colocalization was examined by confocal microscopy. **B.** Surface staining of a2V (red) is demonstrated in cells that were permeabilized or non-permeabilized. Nucleus was stained with DAPI (blue). Scale bars: 10 µm

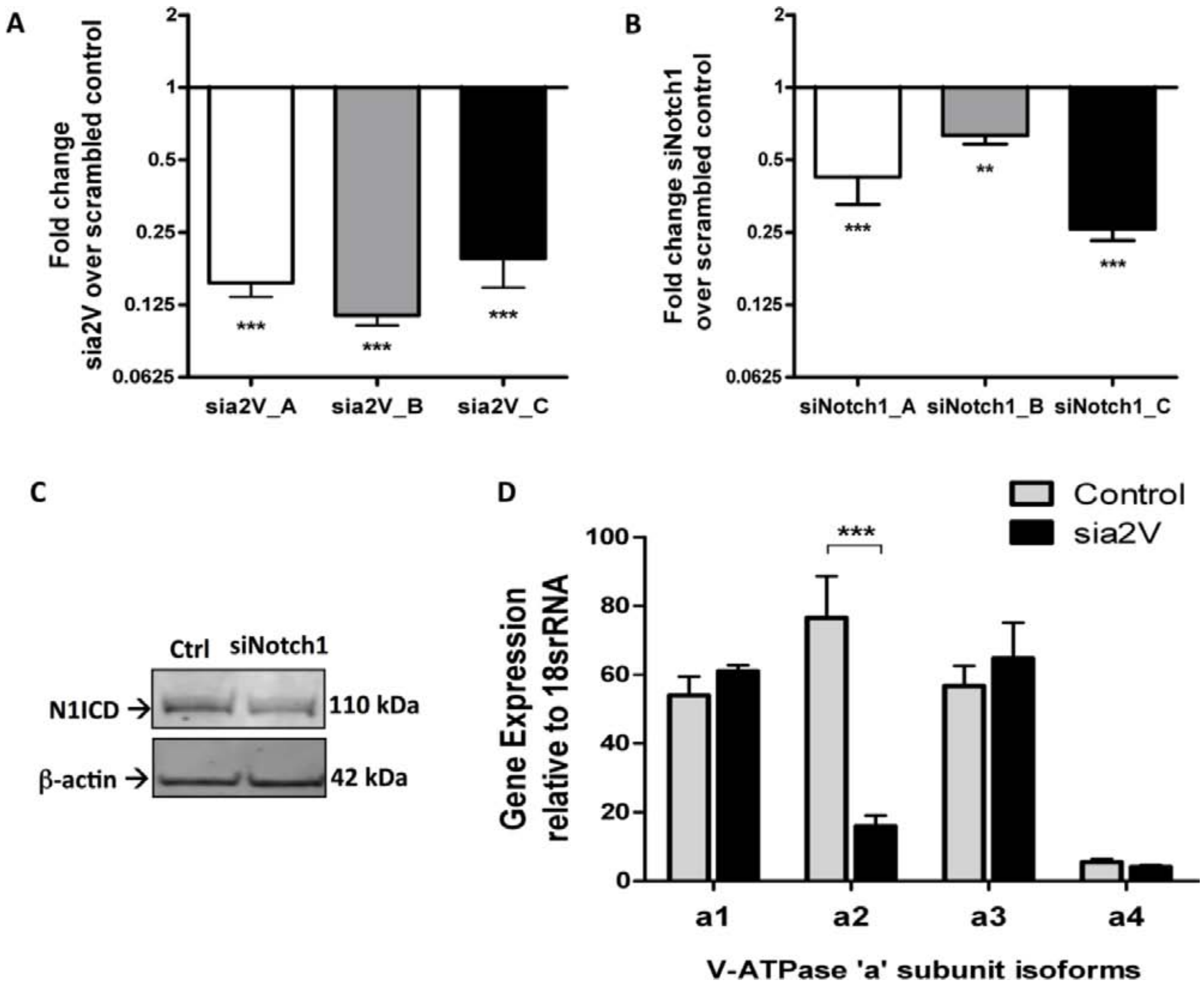

Supplementary Figure S3: Knockdown efficiency of a2V and Notch1 siRNA. MDA-MB-231 cells were seeded in 6 well plates and transfected with three independent siRNA oligonucleotides against a2V or Notch1 along with their scramble control siRNA (10 nM final concentration). Cells were harvested 48 hrs after transfection. Fold change in mRNA expression levels of **A.** a2V and **B.** Notch1 over control siRNA is shown. Prior to fold-change calculation, the values were normalized to the signal generated from endogenous control 18srRNA mRNA. **C.** Knockdown of Notch1 by selected target-specific siRNA was further assessed by western blot. β-actin was used as a loading control. **D.** Effect of a2V gene silencing on mRNA expression levels of other 'a' subunit isoforms of V-ATPase was assessed by qRT PCR. Gene expression relative to 18srRNA is shown. Data represent mean ± standard error, $n = 4$. *$P \leq 0.05$, **$P \leq 0.01$, ***$P \leq 0.001$ compared to control siRNA.

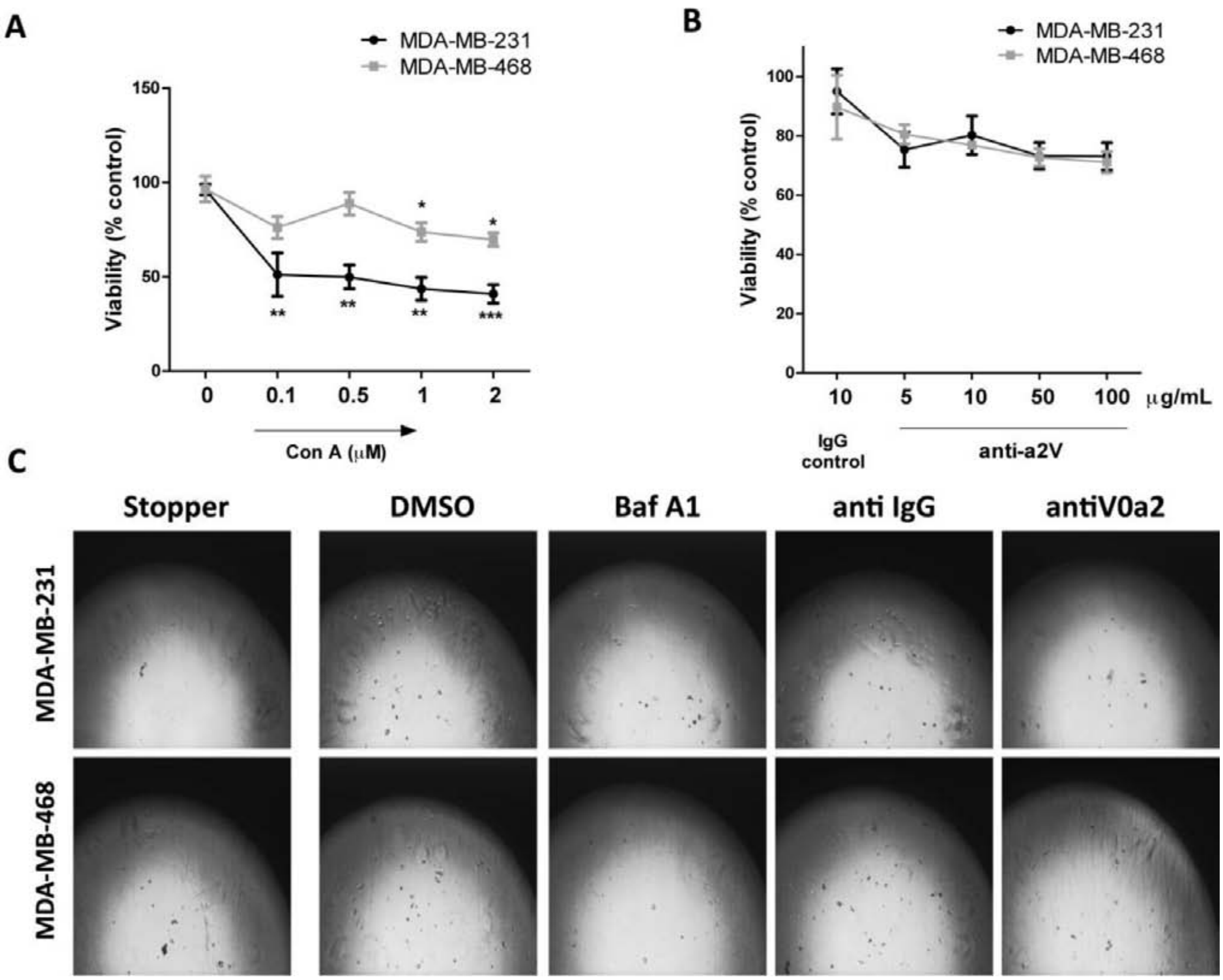

Supplementary Figure S4: Effect of V-ATPase inhibition on TNBC cell viability. A. MDA-MB-231 and MDA-MB-468, cells were seeded (10,000 cells/well) in 96 well plates. Cells were treated with vehicle control or increasing concentrations of (A) Concanamycin A or **B.** anti-a2V for 48 hrs. The viable cell number was assessed by ApoTox-Glo assay and is shown as percent control. Data represent mean ± standard error, $n = 3$. *$P \leq 0.05$, **$P \leq 0.01$, ***$P \leq 0.001$ compared to control. **C.** Representative phase contrast images of the detection zone in cell migration assay of MDA-MB-231 and MDA-MB-468 treated with Baf A1, anti a2V or their representative controls DMSO and IgG.

Supplementary Table S1: Effect of a2V knockdown on genes involved in the Notch Pathway.

Gene Symbol	Name	Mean Fold change sia2V/control	S.E.M
Notch Receptor processing genes			
ADAM17	ADAM metallopeptidase domain 17	1.18	0.18
PSEN1	Presenilin 1	1.45	0.22
Transcription factors and Cofactors			
RBPj	Recombination signal binding protein for immunoglobulin kappa J region	1.17	0.17
MAMl1	Mastermind-like 1 (Drosophila)	1.45	0.22
SWN1	SNW domain containing 1	1.65	0.25
HDAC1	Histone deacetylase 1	0.96	0.15

Following a2V knockdown in MDA-MB-231 cells, mRNA expression levels of genes related to Notch Signaling pathway was assessed by Taqman Notch Signaling pathway PCR array. Fold changes in selected genes are shown. Prior to fold-change calculation, the values were normalized to signal generated from endogenous control 18srRNA. Data represent mean and standard error, $n = 4$.
